# The neutrophil-to-lymphocyte ratio on admission is a good predictor for all-cause mortality in hypertensive patients over 80 years of age

**DOI:** 10.1186/s12872-017-0595-1

**Published:** 2017-06-24

**Authors:** Xiaonan Sun, Leiming Luo, Xiaoqian Zhao, Ping Ye, Ruixue Du

**Affiliations:** 1 Department of Geriatric Cardiology, Chinese People’s Liberation Army General Hospital, 28 Fuxing Road, Beijing, 100853 People’s Republic of China; 2 Department of Cardiology, Chinese People’s Liberation Army 305 Hospital, Beijing, 100000 China

**Keywords:** Neutrophil-to-lymphocyte ratio, Red blood cell distribution, Hypertension, All-cause mortality

## Abstract

**Background:**

Immuno-inflammation plays a major role in the process of hypertension. We aimed to evaluate the association between inflammatory markers, neutrophil-to-lymphocyte ratio (NLR), red cell distribution width (RDW) and all-cause mortality in elderly patients with hypertension.

**Methods:**

A total of 341 hypertensive patients over 80 years of age were included to this study. The NLR and RDW were measured on admission and all the selected patients were followed up for up to 90 days. Kaplan–Meier curves were plotted to evaluate the association between the NLR and the all-cause mortality at follow-up. Using Cox regression models, we investigated the prognostic value of NLR and RDW for all-cause mortality.

**Results:**

Patients with higher quartile of NLR linked to high mortality in hypertensive patients at 90 day after admission (16.47%,13.25%,1.14%,1.17% respectively; χ^2^ = 20.581,*P* = 0.000). Surviving patients had lower RDW (13.61 ± 1.37 VS 14.18 ± 1.38, *p* = 0.041) and NLR (4.97 ± 5.72 VS 7.95 ± 6.88,*p* = 0.011). The receiver operating curve (ROC) of the NLR for all-cause mortality had an area under the curve (AUC) =0.714 (95%CI: 0.629–0.798, *P* = 0.000), with acritical value of 2.97, with sensitivity of 92.6%, and a specificity of 52.5%. The ROC of the RDW to predict all-cause mortality, had an AUC =0.654 (95%CI:0.548–0.761, *P* = 0.008), with acritical value of 13.2%.The Kaplan–Meier curve showed a significant difference between different NLR levels (*p* = 0.002). Multivariate Cox proportional hazard analysis shown 3rd quartile of NLR(RR = 9.646, 95% CI 1.302–34.457, *P* = 0.041) and 4th quartiles(RR = 16.451, 95% CI 2.137–66.643, *P* = 0.007) were found to independently predict all-cause death in hypertensive patients over 80 years of age. Higher rank of NLR was link to higher incidence of all-cause death for such patients.

**Conclusion:**

The findings of the present study demonstrate the potential utility of NLR in risk stratification of elderly patients with hypertension to provide information for clinical treatment strategies.

## What is already known about this topic?

NLR and RDW have been proved to be a good indicator of the prognosis of a variety of diseases, such as cancer, coronary heart disease and pulmonary embolism.

## What does this article add?

Higher NLR levels in hypertensive patients aged over 80 admitted to the hospital are good predictors for all-cause mortality 90 days after admission.

## Background

The role of inflammatory and oxidative stress in cardiovascular diseases has been extensively investigated in multiple studies. Many of them have shown a strong and consistent relationship between cardiovascular diseases and inflammation markers, such as, C-reactive protein (CRP) [1]. Both the neutrophil to lymphocyte ratio (NLR) and the red blood cell distribution (RDW) are novel, inexpensive and easily accessible inflammatory markers which have been shown to be associated with various cardiovascular diseases [2, 3].

The NLR is a ratio of two different yet complementary immune pathways, which serves as a marker of poor general health and physiological stress. Indeed, it has been proved to be a good indicator of the prognosis of a variety of diseases, especially in patients with systemic lupus erythematous (SLE) [4]and cancer [5]. Regarding the RDW, the considerable available evidence suggests that the clinical use of the RDW may be broadened beyond the conventional boundaries of erythrocyte disorders, in particular for assisting the diagnosis and prognosis of patients with acute coronary syndrome (ACS), ischemic cerebrovascular disease, peripheral artery disease (PAD), heart failure (HF) and atrial fibrillation (AF) [6].

Hypertension is one of the most common cardiovascular diseases in the elderly patients. Inflammation and oxidative stress have been implicated in the pathogenesis of hypertension and are the hotspot of hypertension research lately. Patients over 80 years of age are a special group population, usually coming with coronary heart disease, diabetes mellitus (DM), hyperlipidemia, which means that a variety of risk factors superposition together. It was found out there is a certain difference existed between old patients who are aged above 80 and ordinary senile patients indeed through our clinical experience. Such very elderly patients showed differences in the clinical manifestations, treatment response and prognosis and needed more attention. For such kind of patients admitted for all kinds of reasons, whether inflammation markers, such as NLR as well as RDW, can be a predictor for all-cause mortality is still not clear. Accordingly, we designed this preliminary study to investigate the role of these inflammation makers in the evaluation of the prognosis of hypertensive patients.

The aim of this study was to identify independent inflammation predictors of all-cause mortality in hospitalized elderly hypertensive patients, so that such high-risk patients can be identified with sufficient diagnostic accuracy to justify close monitoring or even initiation of secondary prevention hypertension.

## Methods

The current study was conducted at Chinese People’s Liberation Army general hospitals with full ethical approval of the Human Investigation Committee in 2010. In addition, informed consent was obtained from all patients.

This study included patients with hypertension who were diagnosed using the criteria listed in Chinese Hypertension Prevention Guide (2010) [7], hospitalized from January 2011 to December 2013, and aged >80 y. These patients were identified based on previous medical history. Patients with malignant tumors were excluded from the study.

Data on demographic characteristics, such as age, sex, lifestyle (smoking, drinking) and basic medical history, were based on a questionnaire survey of the patients, as well as past medical records. The data collected included history of coronary artery disease (CAD), diabetic mellitus (DM), chronic heart failure, dyslipidemia, chronic kidney disease and anemia. General health parameters, such as height, weight, which were used to calculate body mass index, systolic blood pressure and diastolic blood pressure, resting heart rate, respiratory rate were determined inenrolled patients.

Routine blood tests, including leukocyte, red blood cells, neutrophils (N), lymphocyte (L), red blood cell distribution (RDW), hemoglobin (Hgb), platelet, platelet distribution (PDW) were performed on admission for all the patients. In addition, clinical tests of blood biochemistry, liver and kidney function and inflammatory markers, such as C- reactive protein, were carried out in the Central Laboratory of our hospital.

Since most recent events occurred within 3 months after admission through the review of literature [2] and our clinical experience, we determine that all the included patients were followed up for up to 90 days. The follow-up time was set at 7, 14, 30 and 90 days after admission. All patients were followed up by both telephone interviews and medical record review. The end of the follow-up was the death of all causes. Cause of death was ascertained from the death record, i.e. a legal document including time, site and other information.

Continuous data are expressed as means ± standard deviation. The unpaired Student’s t-test and chi-square test were used for comparisons of continuous and categorical variables, respectively. Kaplan–Meier curves were plotted to evaluate the association between the NLR and the all-cause mortality at follow-up. Univariate and multivariate Cox proportional hazard analysis were used to identify predictors of death. The cut-off point with a maximum combined sensitivity and specificity was selected based on the area under curve (AUC) value from the receiver operator characteristics (ROC) analysis. AP-value of 0.05 or less for the two-sided probability was considered to be statistically significant. All statistical analyses were performed using the SPSS software (SPSS-22.0; IBM Corp., Armonk, NY, USA).

## Results

### Baseline characteristic

A total of 341 cases hypertensive patients were enrolled, including 328 males and 13 females, with average age of 87.43 ± 5.21 (81–102) years. Since the retired army cadres are the main components of the patients for our department, little females were enrolled due to the influence of the restriction. Among the enrolled patients, all patients had been diagnosed with hypertension ranging from 5 to 27 years and had received antihypertensive drug treatment. Additionally, all patients had a history of CAD; 83 patients (24.34%) had a history of myocardial infarction (MI); 29 patients had received stent therapy. Moreover, 67 cases were with chronic heart failure, while 167 cases with DM. 125 patients were with anemia and all of them received therapy, whose hemoglobin levels in the normal range.

### Clinical characteristics according to neutrophil to lymphocyte ratio

The median and interquartile range of NLR was 3.01 and 2.05–6.12, respectively. All patients were grouped in the quartile based on their NLR score as follows: less than2.05, quartile 1, (*n* = 85); between2.05 and2.99, quartile 2 (*n* = 88); between 3.00 and 6.12, quartile 3 (*n* = 83); greater than 6.12, quartile 4 (*n* = 85). Table 1 showed the baseline characteristics of patients in different groups. Age, male gender, smoking history, history of MI and stent implantation, chronic heart failure, type 2 DM (T2DM),anemia and chronic kidney disease exhibited no statistical difference among the groups (*p* > 0.05).Hyperlipidemia incidence showed statistically significant difference (*p* < 0.05). Hemodynamic evaluation indicated that patients in the different quartile also had no statistically significant difference in either systolic blood pressure or diastolic blood pressure (*p* > 0.05). The higher quartile was associated with lower serum total cholesterol, triglyceride, serum iron, ejection fraction and platelet distribution width (PDW)as well as higher platelet/lymphocyte ratio, fasting blood glucose (FBG), CRP and white blood cell (WBC) counts (*p* < 0.05).

**Table 1:** Baseline characteristics of subjects by the quartile of the neutrophil–lymphocyte ratio

	NLR				
	quartile 1	quartile 2	quartile 3	quartile4	*P*
	(NLR < 2.05, *n* = 85)	(NLR 2.05–2.99, *n* = 88)	(NLR 3.00 ~ 6.12, *n* = 83)	(NLR > 6.12, *n* = 85)	
Age (year)	87.94 ± 5.05	86.67 ± 4.43	87.80 ± 5.17	87.25 ± 6.02	0.375
Male (*n*,%)	79 (92.94)	87 (98.86)	79 (95.18)	83 (92.94)	0.180
Smoking history (*n*,%)	29 (34.12)	30 (34.09)	28 (33.73)	36 (42.35)	0.585
Prior MI (*n*, %)	25 (29.41)	20 (22.73)	17 (20.48)	21 (24.71)	0.707
Prior stent (*n*, %)	6 (7.06)	7 (7.95)	9 (10.84)	7 (8.24)	0.837
Heart failure (*n*, %)	16 (18.82)	22 (25.00)	11 (13.25)	18 (21.18)	0.271
Hyperlipidemia (*n*, %)	43 (50.58)	52 (59.09)	48 (57.83)	34 (40.00)	0.049
Chronic kidney diease (*n*, %)	25 (29.41)	25 (28.41)	28 (33.73)	18 (21.18)	0.316
DM (*n*, %)	46 (54.12)	43 (48.86)	43 (51.80)	35 (41.18)	0.357
Anemia (*n*, %)	29 (34.12)	36 (40.91)	28 (33.73)	32 (37.64)	0.724
SBP (mmHg)	130.74 ± 16.17	132.97 ± 19.19	134.61 ± 19.99	132.72 ± 19.03	0.599
DBP (mmHg)	66.78 ± 10.92	69.62 ± 13.55	68.72 ± 12.23	67.61 ± 10.57	0.724
Hypertension with very high risk (*n*, %)	83(97.65)	85(96.59)	80(96.39)	83(97.65)	0.759
BMI (kg/m2)	24.59 ± 3.05	23.96 ± 2.72	24.51 ± 3.33	23.32 ± 3.27	0.079
Hemoglobin (g/l)	123.12 ± 15.64	121.03 ± 18.69	121.93 ± 17.15	125.69 ± 18.77	0.417
WBC (*109/l)	6.20 ± 2.26	6.60 ± 2.34	8.28 ± 3.15	10.89 ± 4.19	0.000
RDW (%)	13.37 ± 0.83	13.82 ± 1.53	13.84 ± 1.38	13.16 ± 1.60	0.084
Platelet (*109/l)	169.92 ± 4.31	178.49 ± 56.76	184.76 ± 71.57	187.54 ± 56.15	0.196
Platelet/lymphocyte ratio	488.36 ± 150.47	694.37 ± 242.26	1086.24 ± 510.12	2669.29 ± 1787.72	0.000
PDW (%)	12.06 ± 1.69	11.57 ± 2.98	10.25 ± 2.86	10.51 ± 3.39	0.005
Creatinine (umol/l)	110.26 ± 53.32	108.89 ± 51.46	113.69 ± 51.07	103.69 ± 57.67	0.646
GFR (ml/min)	72.32 ± 30.42	70.87 ± 26.37	72.21 ± 33.17	79.12 ± 34.55	0.313
FBG (mmol/l)	6.37 ± 2.46	6.11 ± 1.89	7.31 ± 2.56	8.80 ± 2.89	0.000
Fe (umol/l)	17.27 ± 7.16	14.39 ± 8.07	12.18 ± 7.97	8.91 ± 6.02	0.000
TC (mmol/l)	3.08 ± 1.68	2.92 ± 1.76	2.68 ± 1.89	1.79 ± 2.00	0.000
HDL-C (mmol/l)	0.99 ± 0.42	0.98 ± 0.45	1.06 ± 0.42	1.30 ± 0.45	0.001
LDL-C (mmol/l)	1.98 ± 0.83	1.92 ± 0.88	2.04 ± 0.75	1.91 ± 0.73	0.834
TG (mmol/l)	1.33 ± 1.18	1.22 ± 1.08	1.03 ± 1.01	0.84 ± 1.03	0.000
CRP (mg/dl)	0.98 ± 1.78	1.62 ± 2.51	2.34 ± 2.58	4.89 ± 5.69	0.000
Ejection fraction (%)	53.78 ± 11.89	50.72 ± 13.27	41.52 ± 13.30	36.33 ± 8.17	0.000

*DM* Diabetes mellitus, *SBP* Systolic blood pressure, *DBP* Diastolic blood pressure, *BMI* Body mass index, *WBC* White blood cell, *RDW* Red cell distribution width, *PDW* Platelet distribution width, *GFR* Glomerular filtration rate, *FBG* Fasting blood glucose, *TC* Total cholesterol, *TG* Triglyceride, *HDL-C* High density lipoprotein cholesterol, *LDL-C* Low density lipoprotein cholesterol, *CRP* C-reactive protein

Enrolled patients were also grouped in the quartile based on their RDW score as follows: less than 12.8, quartile 1, (*n* = 89); between 12.8 and 13.4, quartile 2 (*n* = 89); between 13.4 and 14.2, quartile 3 (*n* = 95); greater than 14.2, quartile 4 (*n* = 88) and Table 2 showed the baseline characteristics of subjects in different groups. History of chronic heart failure, anemia and chronic kidney disease exhibited statistically significant difference (*p* < 0.05). The higher quartile RDW level was associated with lower serum hemoglobin, ejection fraction (*p* < 0.05). PDW also showed difference among groups but its trend cannot be determined.

**Table 2:** Baseline characteristics of subjects by the quartile of red cell distribution width

	RDW				
	quartile 1	quartile 2	quartile 3	quartile4	P
	(RDW < 12.8,*n* = 69)	(RDW 12.8–13.3,*n* = 89)	(RDW 13.4 ~ 14.2,*n* = 95)	(RDW > 14.2,*n* = 88)	
Age (year)	87.25 ± 4.76	86.31 ± 5.43	88.46 ± 5.25	87.55 ± 4.92	0.067
Male (*n*, %)	67 (97.10)	85 (95.51)	94 (98.95)	82 (93.18)	0.210
Smoking history (*n*, %)	23 (33.33)	35 (39.33)	34 (35.79)	31 (35.23)	0.882
Prior MI (*n*, %)	20 (28.99)	20 (22.47)	22 (23.15)	24 (27.27)	0.166
Prior stent (*n*, %)	7 (10.14)	11 (12.36)	3 (3.15)	8 (9.10)	0.099
Heart failure (*n*, %)	7 (10.16)	17 (19.10)	17 (17.89)	26 (29.55)	0.022
Hyperlipidemia (*n*, %)	38 (55.07)	39 (43.82)	44 (46.31)	56 (63.64)	0.034
CKD (*n*, %)	14 (20.21)	23 (25.83)	24 (25.26)	36 (40.91)	0.021
DM (*n*, %)	38 (55.07)	44 (49.43)	47 (49.47)	38 (43.18)	0.527
Anemia (*n*, %)	15 (21.73)	26 (29.21)	39 (41.05)	45 (51.10)	0.011
SBP(mmHg)	132.52 ± 18.27	132.17 ± 18.43	131.28 ± 19.04	135.21 ± 18.72	0.529
DBP (mmHg)	70.56 ± 15.08	61.62 ± 10.51	68.52 ± 11.19	65.71 ± 10.95	0.062
Hypertension with very high risk (*n*, %)	67 (97.10)	85 (95.50)	92 (96.84)	87 (98.86)	0.778
BMI (kg/m2)	24.73 ± 3.17	23.77 ± 2.86	23.91 ± 3.21	24.26 ± 3.15	0.300
Hemoglobin (g/l)	128.79 ± 13.67	125.04 ± 16.80	122.29 ± 16.87	117.52 ± 19.77	0.010
WBC (*109/l)	7.67 ± 3.78	7.37 ± 3.21	8.15 ± 3.53	8.75 ± 3.75	0.061
Platelet (*109/l)	173.83 ± 49.55	179.10 ± 52.42	180.41 ± 52.30	186.13 ± 72.84	0.612
PDW (%)	10.60 ± 3.42	11.27 ± 2.48	11.88 ± 1.73	11.43 ± 1.63	0.049
Creatinine (umol/l)	96.29 ± 28.91	102.37 ± 34.73	109.86 ± 34.74	125.03 ± 72.26	0.040
GFR (ml/min)	78.48 ± 29.16	74.02 ± 26.46	72.51 ± 32.65	68.50 ± 35.67	0.252
FBG (mmol/l)	7.26 ± 2.83	6.83 ± 2.51	6.98 ± 2.50	7.01 ± 2.57	0.787
Fe (umol/l)	13.73 ± 8.23	15.09 ± 9.53	12.15 ± 6.06	11.91 ± 7.41	0.029
TC (mmol/l)	2.87 ± 2.00	2.83 ± 1.78	2.50 ± 1.97	2.53 ± 1.87	0.621
HDL-C (mmol/l)	1.05 ± 0.41	1.14 ± 0.43	1.01 ± 0.48	1.04 ± 0.45	0.319
LDL-C (mmol/l)	2.14 ± 0.97	1.97 ± 0.64	1.91 ± 0.95	1.92 ± 0.62	0.387
TG (mmol/l)	1.08 ± 1.18	0.99 ± 0.90	0.91 ± 1.00	1.16 ± 136	0.477
CRP (mg/dl)	2.33 ± 4.75	2.80 ± 3.18	2.78 ± 4.30	2.85 ± 3.71	0.900
Ejection fraction(%)	48.70 ± 13.61	46.61 ± 13.73	44.93 ± 14.25	42.29 ± 12.30	0.023

*RDW* Red cell distribution width, *DM* Diabetes mellitus, *CKD* Chronic kidney disease, *SBP* Systolic blood pressure, *DBP* Diastolic blood pressure, *BMI* Body mass index, *WBC* White blood cell, *RDW* Red cell distribution width, *PDW* Platelet distribution width, *GFR* Glomerular filtration rate, *FBG* Fasting blood glucose, *TC* Total cholesterol, *TG* Triglyceride, *HDL-C* High density lipoprotein cholesterol, *LDL-C* Low density lipoprotein cholesterol, *CRP* C-reactive protein

### Follow-up results

In total, 27 death were recorded in 90-day follow-up and most took place between the 30 and 90 day (*n* = 17, 62.97%) after admission. The association between the various quartiles and mortality is presented in Table 3. Patients with the higher quartile are linked to high mortality (16.47%, 13.25%, 1.14%, 1.17% respectively; χ^2^ = 20.581, *P* < 0.001). The parameter and characteristics of different outcomes of the patients are shown in Table 4. Surviving patients had a higher BMI (24.25 ± 3.05 VS 24.25 ± 3.05, *p* = 0.012) and hemoglobin (123.78 ± 17.05 VS 115.07 ± 20.42, *P* = 0.040), as well as lower DBP (62.48 ± 9.60 VS 68.31 ± 12.02, *p* = 0.016), RDW (13.61 ± 1.37 VS 14.18 ± 1.38, *p* = 0.041), platelet lymphocyte ratio (1184.80 ± 1235.71 VS 1836.84 ± 1416.97, *p* = 0.010) and NLR (4.97 ± 5.72 VS 7.95 ± 6.88, *p* = 0.011).

**Table 3:** All-cause death in different quartile

Group	All-cause death			
	Day 7	Day 14	Day 30	Day 90
quartile1 (*n*, %)	1 (1.18)	0 (0.00)	0 (0.00)	0 (0.00)
quartile2 (*n*, %)	0 (0.00)	0 (0.00)	1 (1.14)	0 (0.00)
quartile3 (*n*, %)	0 (0.00)	1 (1.14)	2 (2.41)	8 (9.41)
quartile4 (*n*, %)	2 (2.35)	1 (1.14)	2 (2.41)	9 (10.59)

**Table 4:** Comparison of the laboratory parameters of different outcome

	Death for all cause	Survival	*P*
	(*n* = 27)	(*n* = 314)	
Age (year)	89.29 ± 4.57	87.26 ± 5.25	0.052
Prior MI (*n*, %)	7 (25.93)	79 (25.16)	0.943
Heart failure (*n*,%)	3 (11.11)	64 (20.38)	0.318
DM (n,%)	15 (55.56)	152 (48.41)	0.304
Hyperlipidemia (*n*, %)	12 (44.44)	165 (52.55)	0.431
BMI (kg/m^2^)	22.31 ± 3.31	24.25 ± 3.05	0.012
SBP(mmHg,	133.29 ± 18.43	126.85 ± 20.16	0.085
DBP (mmHg)	68.31 ± 12.02	62.48 ± 9.60	0.016
Hemoglobin (g/l)	115.07 ± 20.42	123.78 ± 17.05	0.040
WBC (*10^9^/l)	8.90 ± 5.62	7.92 ± 3.35	0.176
RDW (%)	14.18 ± 1.38	13.61 ± 1.37	0.041
Platelet (*10^9^/l)	188.07 ± 60.65	179.55 ± 57.54	0.463
Platelet/lymphocyte ratio	1836.84 ± 1416.97	1184.80 ± 1235.71	0.010
NLR	7.95 ± 6.88	4.97 ± 5.72	0.011
Creatinine (umol/l)	110.74 ± 61.19	108.97 ± 52.44	0.868
CRP (mg/dl)	3.49 ± 2.88	2.63 ± 4.08	0.316

*MI* Myocardial infarction, *DM* Diabetes mellitus, *SBP* Systolic blood pressure, *DBP* Diastolic blood pressure, *BMI* Body mass index, *RDW* Red cell distribution width, *CRP* C-reactive protein

**Fig. 1:**
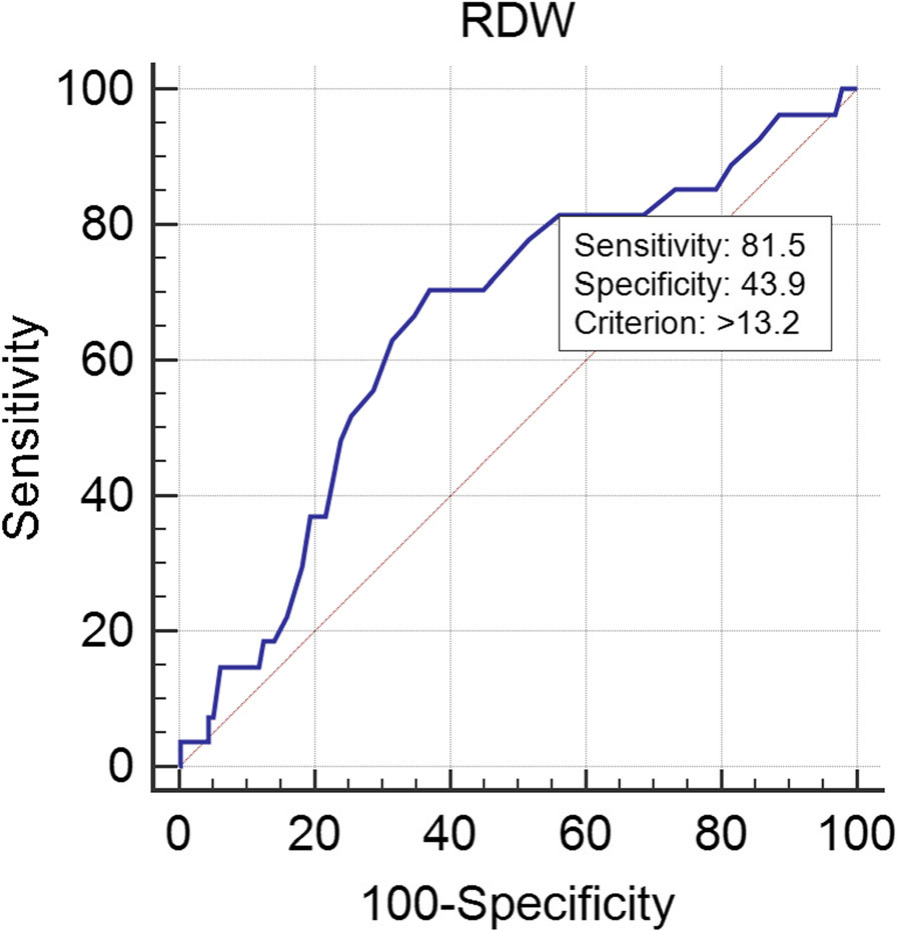
ROC curve of the RDW

Mapping the ROC of the RDW to predict all-cause mortality (Fig. 1), with an AUC = 0.654 (95% CI: 0.548–0.761, *P* = 0.008), a critical value of13.2%, a corresponding predictive sensitivity of 81.5% and a specificity of 43.9%.

**Fig. 2:**
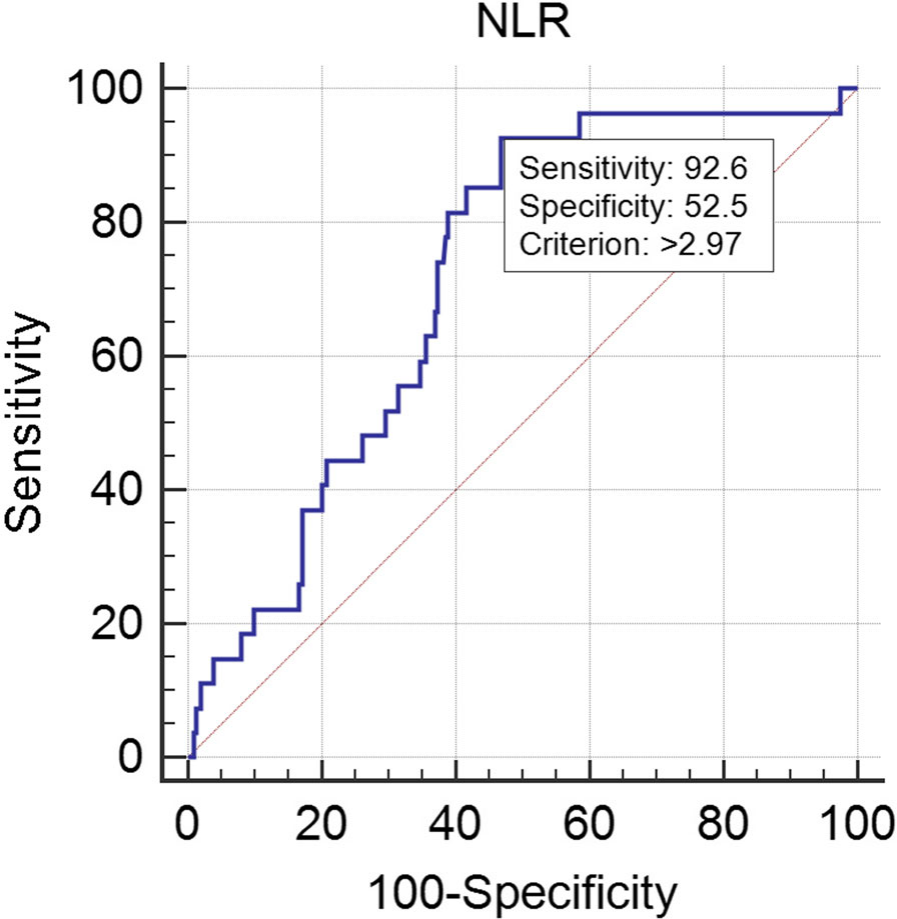
ROC curve of the NLR

The ROC for the NLR is shown in Fig. 2, it had an AUC = 0.714 (95%CI: 0.629–0.798, *P* < 0.001), the critical value was 2.97, with a sensitivity of 92.6%, and a specificity of 52.5%.

**Fig. 3:**
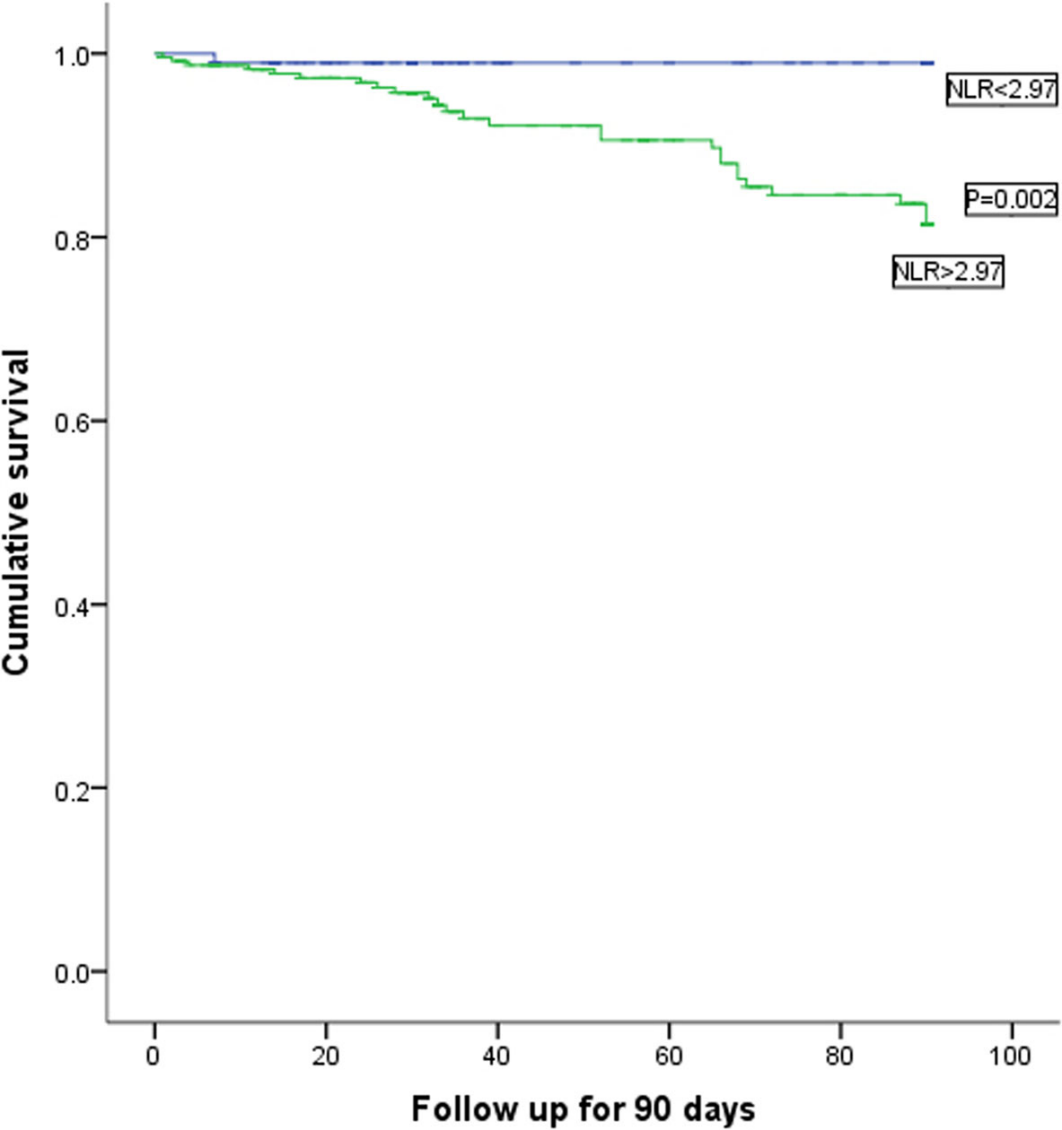
The Kaplan–Meier survival curves for different NLR levels

The Kaplan–Meier curve showed a significant difference in the all-cause mortality between the different NLR levels (Fig. 3). Admission NLR higher than 2.97 were found to be connected with high mortality compared with those less than 2.97 (1.02% VS 10.70%, *p* = 0.002).

**Fig. 4:**
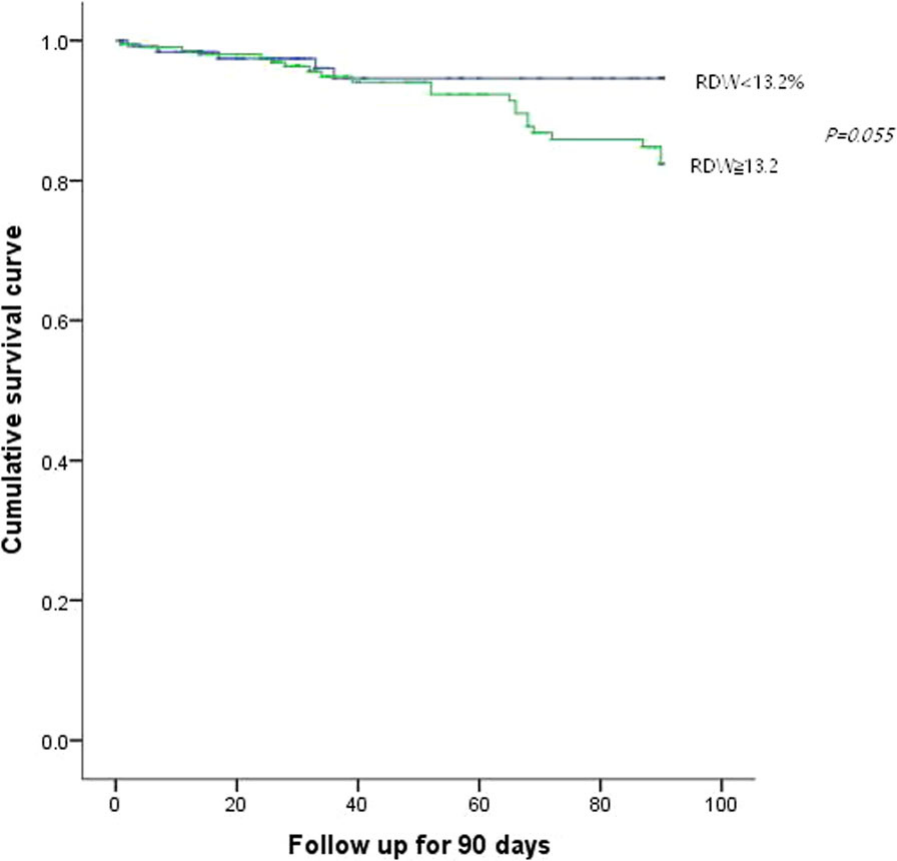
The Kaplan–Meier survival curves for different RDW levels

The Kaplan–Meier curve of RDW showed there is no significant difference in the all-cause mortality between the different RDW levels (χ^2^ = 3.680, *P* = 0.055, Fig. 4).

Univariate and multivariate Cox proportional hazard analyses were performed to investigate the possible predictors of all-cause mortality in the study population (Table 5). It is worth noting that the correlation between the CPR levels and all-cause mortality in such patients is not significant (RR = 1.042, 95% CI 0.895–1.213, *P* = 0.599). On the multivariate Cox proportional hazard analysis, using the lowest NLR quartile as reference, 3rd quartiles (RR = 9.646, 95% CI 1.302–34.457, *P* = 0.041) and 4th quartiles (RR = 16.451, 95% CI 2.137–66.643, *P* = 0.007) were found to independently associate with all-cause death in hypertensive patients over 80 years of age, though 2nd quartile (RR = 3.629, 95% CI 0.373–18.995, *P* = 0.838) was not. Higher rank of NLR is link to higher incidence of all-cause death for such patients.

**Table 5:** Multivariate Cox hazard model NLR for the possible predictors of all-cause death in the study population

	HR	P	IC.inf	IC.sup
Quartile 2	3.629	0.838	0.377	18.995
Quartile 3	9.646	0.041	1.302	34.457
Quartile 4	16.451	0.007	2.137	66.643
SBP	0.993	0.678	0.962	1.025
DBP	0.956	0.096	0.906	1.008
BMI	0.898	0.190	0.765	1.055
Age	1.126	0.053	0.998	1.271
CRP	1.042	0.599	0.895	1.213

*RDW* Red cell distribution width, *SBP* Systolic blood pressure, *DBP* Diastolic blood pressure, *BMI* Body mass index, *CRP* C-reactive protein, *HR* hazard ratio.

Cox proportional hazard analyses were also performed for RDW and the results were shown in Table 6. Increased relative risk was not found among different RDW quartile groups (RR = 0.737, 95% confidence interval 0.111–4.883, *P* = 0.752; RR = 1.872, 95% CI 0.811–8.252, *P* = 0.088 and RR = 2.588, 95% CI 0.096–10.102, *P* = 0.053, in 2nd, 3rd, 4th quartile groups respectively).

**Table 6:** Multivariate Cox hazard model of RDW for the possible predictors of All-cause death in the study population

	HR	P	IC.inf	IC.sup
RDW Quartile 2	0.737	0.752	0.111	4.883
RDW Quartile 3	1.872	0.088	0.811	8.252
RDW Quartile 4	2.588	0.053	0.960	10.012
SBP	0.982	0.315	0.949	1.017
DBP	0.972	0.330	0.918	1.029
BMI	0.901	0.266	0.750	1.083
Age	1.094	0.163	0.965	1.240
CRP	1.113	0.117	0.973	1.273

*RDW* Red cell distribution width, *SBP* Systolic blood pressure, *DBP* Diastolic blood pressure, *BMI* Body mass index, *CRP* C-reactive protein, *HR* hazard ratio

## Discussion

The main finding of the present study was that elevated NLR on admission was an independent predictor of all-cause mortality in 90 day for hospitalized hypertensive patients over the age of 80. With the increase of NLR, the incidence of all-cause death increased (3rd quartiles: RR = 9.646, *P* = 0.041and 4th quartiles: RR = 16.451, *P* = 0.007). Regarding the ROC analysis, an admission NLR higher than 2.97 was found to predict all-cause mortality with a sensitivity of 92.6% and a specificity of 52.5% (AUC = 0.714, *P* = 0.00). To our knowledge this is the first report of such findings in such patient population.

Inflammation and oxidative stress have been implicated in the pathogenesis of cardiovascular disease, and thus inflammatory biomarkers have received considerable attention. RDW and NLR have recently emerged as potential new biomarkers that discriminate individuals at risk for future adverse events in patients with cardiovascular disease, which were both inexpensive and easily accessible [8, 9]. Simple indicators that can provide a wealth of information for clinical hypertension deserve more attention.

Indeed, increased RDW was found to be an independent predictor of mortality in patients with heart failure [10], further studies showed that the RDW is a prognostic indicator for patients with HF caused by coronary heart disease and dilated cardiomyopathy [11]. The RDW is also an independent predictor of the coronary artery calcification, suggesting that it might be a useful marker for predicting CAD [12]. Elevated RDW levels, may be an independent risk marker for non-valvular AF [13]. Accordingly, the RDW is considered to be a good indicator of the prognosis of cardiovascular diseases, but few studies have been carried out in hypertensive patients. Sarikaya et al. [14] found that the RDW levels were higher in hypertensive patients with AF. An increased RDW level in such patients with hypertension may alert physician on the development or presence of AF. Tanindi et al. [15] found that higher RDW values are strongly correlated with higher systolic and diastolic blood pressures. But in our study revealed that, though the RDW level showed difference between surviving and death, Cox proportional hazard analyses indicated higher RDW level wasn’t accompanied with an increased risk of all-cause mortality 90 days after admission.

Unlike many other inflammatory markers and bioassays, the NLR is an inexpensive and readily available marker which is a combination of two independent markers of inflammation, providing us with additional information. It also can be an index for of sympathetic/parasympathetic tone balance. A higher level of NLR could indicate a higher ratio of sympathetic/parasympathetic tone [16]. So it may provide us with more information in cardiovascular disease. Recently, many studies focused on the NLR and their association with adverse outcomes in patients with cardiovascular disease, but most of these studies focused on CAD or heart failure, especially in patients with ACS. High NLR levels, white blood cell counts, and neutrophil counts at admission are independently correlated with stent restenosis after primary PCI [17, 18]. In ST elevation myocardial infarction patients, the frequency of ventricular tachyarrhythmia (VT/VF) at the first day was associated with higher neutrophil count (*P* < 0.001) and higher NLR level (*P* < 0.001) [19]. Average NLR was a useful and powerful predictor of mortality and adverse-outcomes in Chinese patients presenting with ST segment elevation myocardial infarction [20]. Benites et al. [21]found intermediate and high NLR tertiles remained significantly associated with all-cause mortality (HR = 1.83, 95% CI 1.07 to 3.14 and HR = 2.16, 95% CI 1.21 to 3.83) in advanced heart failure. Cut-off value of 5.1 for NLR could predict death in HF patients with 75% sensitivity and 62% specificity during a 12.8-month follow-up period on average, thus NLR was considered to be used to predict mortality during the follow-up of HF patients [22].

Fewer studies have focused on NLR and hypertension. A study on resistance hypertension showed that NLR level was increased in hypertension patients and the resistance hypertension group had a significantly higher NLR than the control hypertension group (*P* = 0.03) [23]. Hypertensive patients with high homocysteine(HCY) had increased NLR and it positively correlated with HCY but not with blood pressure [24]. Also, there was a statistically significant positive correlation between the ascending aortic diameter and NLR (*r* = 0.524, *P* < 0.001), which indicated that it plays a role in the pathogenesis of aneurysm of the ascending aorta in hypertensive patients [25]. In addition, hypertensive patients with diastolic dysfunction had higher values of NLR compared with subjects without diastolic dysfunction. Furthermore higher grades of diastolic dysfunction were associated with higher levels of NLR [26]. And patients with non-dipper hypertension had significantly higher NLR and PLR compared to dipper hypertension, which has not been reported previously [27]. All these findings suggest that higher NLR values may link to poor clinical outcome in subtypes of hypertension, which make us believed that NLR could be used for risk stratification and it may be a good predictor for the prognosis of hypertension. Now our research first confirms such points. We found that a higher quartile of NLR was tend to have a higher incidence of death, and increased NLR quartile will increased the risk of all-cause death (3rd quartiles: RR = 9.646, 95% confidence interval 1.302–34.457, *P* = 0.041and 4th quartiles:RR = 16.451, 95% confidence interval 2.137–66.643, *P* = 0.007). So we believed that high NLR level was an independent predictor of all-cause mortality in hypertensive patients over 80 years of age, which provided us convenient and preliminary screening tool.

NLR can be easily calculated from complete blood cell count performed in nearly every patient. With this knowledge, NLR may lead physicians to identify high risk patients who require closer care because of increased risks of all-cause death easily. And will help us to use the medical resources more efficient. Together with other index, the specificity may improve. Our results may provide a simple and effective tool of preliminary screening for such kind of patients, which is the most important value of research. We also see that NLR have high sensitivity but low specificity, which hint us that high NLR level is just a preliminary tool and it may be necessary to combine with other indicators to provide more accurate evaluation information.

The limitations of the present study are as follows. (1) This was a single center study that included a relatively small number of patients. (2) Only one measurement of admission full blood count and calculation of RDW and NLR was included in the analysis.

## Conclusion

We found that higher NLR levels in hypertensive patients admitted to the hospital are good predictors for all-cause mortality 90 days after admission. This indicated that NLR, which is easily determinable, broadly available and inexpensive markers, could be used to identify patients at high risk for adverse endpoints. However, these findings must be confirmed on a study with a larger patient population.

## Abbreviations

ACS: Acute coronary syndrome
AF: Atrial fibrillation
AUC: Area under curve
BMI: Body mass index
CAD: Coronary artery disease
CRP: C-reactive protein
DBP: Diastolic blood pressure
DM: Diabetes mellitus
FBG: Fasting blood glucose
GFR: Glomerular filtration rate
HCY: Homocysteine
HDL-C: High density lipoprotein cholesterol
HF: Heart failure
Hgb: Hemoglobin
LDL-C: Low density lipoprotein cholesterol
NLR: Neutrophil-to-lymphocyte ratio
PAD: Peripheral artery disease
PDW: Platelet distribution width
RDW: Red cell distribution width
ROC: Receiver operator characteristics
SBP: Systolic blood pressure
SLE: Systemic lupus erythematosus
TC: Total cholesterol
TG: Triglyceride
